# The subperitoneal space and peritoneal cavity: basic concepts

**DOI:** 10.1007/s00261-015-0429-5

**Published:** 2015-05-26

**Authors:** Harpreet K. Pannu, Michael Oliphant

**Affiliations:** Department of Radiology, Memorial Sloan Kettering Cancer Center, 1275 York Avenue, New York, NY 10065 USA; Department of Radiology, Wake Forest University School of Medicine, Winston-Salem, NC USA

**Keywords:** Subperitoneal space, Peritoneal cavity, Anatomy

## Abstract

The subperitoneal space and peritoneal cavity are two mutually exclusive spaces that are separated by the peritoneum. Each is a single continuous space with interconnected regions. Disease can spread either within the subperitoneal space or within the peritoneal cavity to distant sites in the abdomen and pelvis via these interconnecting pathways. Disease can also cross the peritoneum to spread from the subperitoneal space to the peritoneal cavity or vice versa.

The article is based on the comprehensive and authoritative book by Drs Meyers, Charnsangavej, and Oliphant titled Meyers’ Dynamic Radiology of the Abdomen [1]. Key concepts from the book are highlighted in the following text and in Table 1. The text and accompanying illustrative images are divided into 3 sections on anatomy, an overview of the spread of disease, and disease spread for selected organs.

**Table 1:** Key concepts regarding the peritoneal cavity and subperitoneal space (SPS)

Concept	Discussion
Spaces of the abdomen and pelvis	There are 2 mutually exclusive spaces—the peritoneal cavity and the SPS
The structure that defines the SPS & separates it from the peritoneal cavity	The peritoneum
The peritoneal cavity	The peritoneal cavity is a potential space and is inconspicuous on normal imaging studies. There are no organs in the peritoneal cavity.
The SPS	The SPS is a single extraperitoneal space encompassing the entire abdomen and pelvis. It is divided into regions but remains one interconnected space. The SPS contains all the organs, vessels, lymphatics, and nerves of the abdomen and pelvis.
The importance of distinguishing the SPS from the peritoneal cavity	The routes of disease spread differ for the SPS and the peritoneal cavity
The distinct patterns of disease spread in the SPS	Disease spread can occur via mesenteries, ligaments, and lymphatics, and by periarterial, perineural, transvenous, and intratubular routes
The distinct patterns of disease spread within the peritoneal cavity	Disease spreads along the pathways of fluid flow in the single continuous space of the peritoneal cavity
Transperitoneal disease spread	Transperitoneal spread occurs when disease crosses the peritoneum which separates the SPS from the peritoneal cavity. Transperitoneal spread is bidirectional

## Anatomy

### Lack of organs in the peritoneal cavity

The peritoneum is a serous membrane made up of visceral and parietal layers. The visceral layer of the peritoneum lines the surface of organs and the parietal peritoneum lines the coelomic cavity. The peritoneal cavity is a potential space between the visceral and parietal layers of the peritoneum. There are no organs in the peritoneal cavity. The potential space of the peritoneal cavity is normally not visible on imaging as it contains only a small amount of fluid (about 100 mL). The peritoneum is analogous to the pleura which has a visceral layer covering lung and a parietal layer lining the thoracic cavity. Similar to the pleural cavity, the peritoneal cavity is visualized on imaging if it is abnormally distended by fluid, gas, or masses.

### Location of the abdominal and pelvic organs

**Fig. 1:**
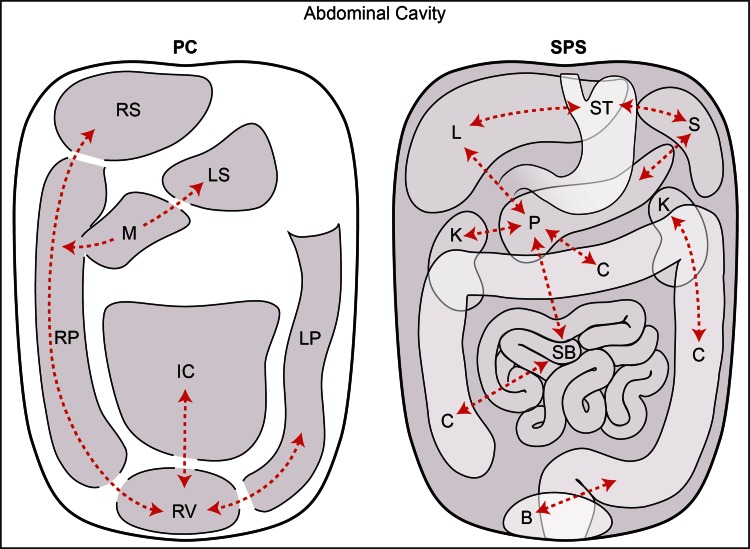
The peritoneal cavity vs the subperitoneal space. This is a schematic diagram showing the peritoneal cavity (PC) on the *left* and the subperitoneal space (SPS) on the *right*. The diagram illustrates that these are 2 completely separate spaces within the abdominal cavity. The peritoneal cavity and the subperitoneal space are separated by the peritoneum. The peritoneal cavity is one continuous space with interconnecting recesses, some of which are shown. The subperitoneal space is also one continuous space containing all the abdominal pelvic organs which are interconnected via ligaments and mesenteries. *Dotted lines* show some of these interconnections which allow for disease spread. Abbreviations for the peritoneal cavity: *IC* inframesocolic compartment, *LP* left paracolic recess, *LS* lesser sac, *M* Morison’s pouch, *RP* right paracolic recess, *RS* right subphrenic space, and *RV* rectovesical space. Abbreviations for the subperitoneal space: *B* bladder, *C* colon, *K* kidney, *L* liver, *P* pancreas, *S* spleen, *SB* small bowel, and *ST* stomach.

**Fig. 2:**
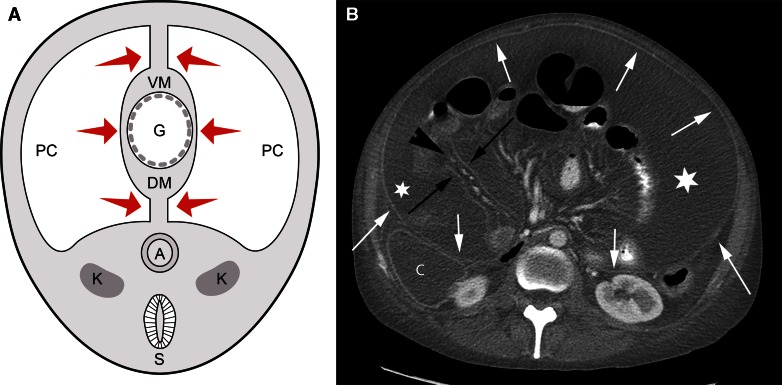
Relationship between the mesentery and the peritoneum. **A** Diagram of a 4-week-old embryo shows the coelomic cavity which will form the peritoneal cavity (PC) surrounding the primitive gut (G). The medial visceral layer (*arrows*) of the peritoneum is apposed on the gut and the mesentery, while the parietal layer is lateral. The dorsal mesentery (DM) conveys vessels from the aorta to the gut. Other than the peritoneal cavity, all the tissue in the abdominal cavity portion of the diagram is the subperitoneal space (*light gray shaded area*). Spine is shown on the diagram only for orientation purposes. *A* aorta, *K* kidney, *S* spine, and *VM* ventral mesentery. **B** Axial CT image shows the dorsal mesentery of the small bowel as subperitoneal tissue (*arrowhead*) between 2 layers of visceral peritoneum (*black arrows*). The parietal peritoneal reflection (*white arrows*) is also seen anterior to the colon (C), kidney, and in the anterior and lateral abdomen. There is fluid (*asterisk*) in the peritoneal cavity between the visceral (*black arrows*) and parietal (*white arrows*) layers of the peritoneum.

**Fig. 3:**
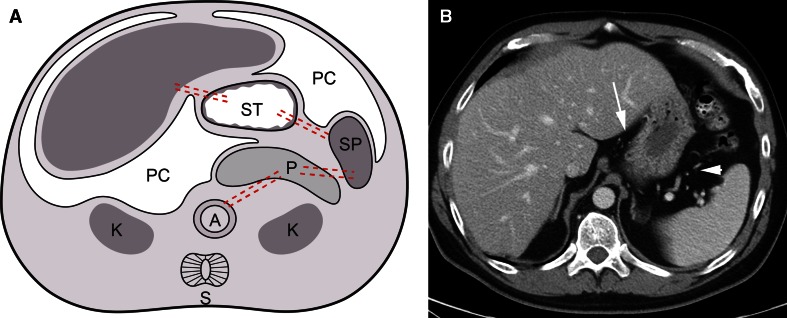
Abdominal ligaments. **A** Diagram showing development of an embryo. The liver develops in the ventral mesentery anterior to the stomach. The residual part of the ventral mesentery between the liver and stomach is called the gastrohepatic ligament in the adult. The spleen and pancreas form in the dorsal mesentery posterior to the stomach. The residual part of the dorsal mesentery between the spleen and stomach is called the gastrosplenic ligament in the adult. The pancreas fuses with the tissues anterior to the kidney to lie in the anterior pararenal space. The residual part of the dorsal mesentery between the spleen and pancreas is called the splenorenal ligament. *Dotted lines* approximate the paths of the ventral and dorsal mesenteries. *A* aorta, *K* kidney, *L* liver, *PC* peritoneal cavity, *S* spine, and *ST* stomach. Subperitoneal space = light gray shaded area in abdominal cavity portion of diagram. Spine is shown on the diagram only for orientation purposes. **B** Axial CT image of the upper abdomen shows the gastrohepatic (*arrow*) and gastrosplenic (*arrowhead*) ligaments containing the left gastric artery and short gastric vessels, respectively.

There are two spaces in the abdomen and pelvis, the peritoneal cavity (a potential space) and the subperitoneal space, and these are separated by the peritoneum (Fig. 1). Regardless of the complexity of development in the embryo, the subperitoneal space and the peritoneal cavity remain separated from each other, and each remains a single continuous space (Figs. 2A, 3A). Distinguishing the subperitoneal space from the potential space of the peritoneal cavity is important for understanding the distinct patterns of disease spread in each.

The term subperitoneal refers to tissue that is deep to the peritoneum and includes the extraperitoneal space, the ligaments and the mesenteries and their suspended organs (Fig. 2A). Organs whose surfaces are covered by peritoneum are therefore subperitoneal. Subperitoneal organs that are deep to the posterior peritoneum are called extraperitoneal. Since there are only 2 spaces in the abdomen and there are no organs in the peritoneal cavity, all the abdominal pelvic organs, and their associated vessels, lymphatics, and nerves are in the subperitoneal space. In other words, all the structures seen in the abdomen and pelvis on cross-sectional imaging are in the subperitoneal space. The organs lie in the abdominal cavity, not the peritoneal cavity (Figs. 1, 2A). The peritoneal cavity is a potential space devoid of organs.

### Importance of visualizing the subperitoneal space as a single space

The subperitoneal space is a large continuous space that is formed by regions interconnected by ligaments and mesenteries. Ligaments and mesenteries refer to the subperitoneal tissue between suspended organs and the extraperitoneal space. Visualizing the subperitoneal space as a single space explains the spread of disease between different regions of the abdomen pelvis and between the organs covered by peritoneum and the extraperitoneum.

### The relationship between the bowel mesentery and the peritoneum

The mesenteries of the abdomen and pelvis are composed of subperitoneal tissue between 2 layers of visceral peritoneum. Comparing an axial CT image with a cross-sectional diagram of an embryo, the dorsal mesentery carries vessels from the aorta to the gut (Fig. 2). The visceral peritoneum surrounds the mesentery, forms the serosal layer of the gut, and is in continuity with the parietal peritoneum which covers the extraperitoneal space. As the ligaments, mesenteries, and suspended organs develop, the peritoneal cavity forms recesses that remain interconnected as the peritoneal cavity and separate from the subperitoneal space (Fig. 3A).

### Correlation between embryonic development and the abdominal ligaments

The spleen, pancreas, liver, and gut form within the mesentery that surrounds and suspends the primitive gut in the embryo. The development of these organs results in the creation of the abdominal ligaments that can be identified on CT.

Posteriorly, the spleen and pancreas form in the dorsal mesogastrium, or the part of the dorsal mesentery suspending the stomach (Fig. 3). The splenic artery runs from the aorta through the dorsal mesogastrium to the spleen with branches continuing to the stomach. The part of the dorsal mesogastrium between the stomach and the spleen becomes the gastrosplenic ligament containing the short gastric vessels. The part of the dorsal mesogastrium containing the pancreas fuses with the subperitoneal tissues anterior to the kidney leaving a connecting splenorenal ligament that contains the distal splenic artery and vein. The midgut and hindgut form within the dorsal mesentery creating the small intestine mesentery and the mesocolon.

Anteriorly, the liver forms in the subperitoneal tissues anterior to the stomach, or the ventral mesogastrium. This divides the subperitoneal tissues into the gastrohepatic ligament between the stomach and liver, and the falciform ligament between the liver and abdominal wall. The free edge of the gastrohepatic ligament is the hepatoduodenal ligament. The visceral peritoneum continues over the stomach forming the serosal layer and over the liver and spleen forming their capsules.

### Identifying the ligaments and bowel mesenteries on imaging

The ligaments and mesenteries are named according to the viscera they connect and are identified by the vessels that run in them. For example, the hepatoduodenal ligament is identified by the portal vein, hepatic artery, and common bile duct. Selected ligaments and their associated vessels are described in Table 2. The ligaments and mesenteries are a pathway for disease spread between organs. The vasculature within the mesenteries often acts as a scaffold for disease spread. The utility of identifying the mesenteries and ligaments is to more accurately and efficiently recognize sites of disease spread.

**Table 2:** Selected abdominal and pelvic ligaments and mesenteries

Ligament	From	To	Identified By
Hepatoduodenal	Hepatic hilum	Duodenum	Portal vein, hepatic artery
Gastrohepatic (lesser omentum)	Lesser curvature of stomach	Fissure of ligamentum venosum	Left and right gastric artery, replaced left hepatic artery
Gastrosplenic	Greater curvature of proximal stomach	Splenic hilum	Left gastroepiploic and short gastric vessels
Gastrocolic	Greater curvature of gastric body	Transverse colon	Right and left gastroepiploic vessels
Splenorenal	Left anterior pararenal	Splenic hilum	Splenic vessels near hilum
Small bowel mesentery root	Duodenal-jejunal junction	Right iliac fossa	Superior mesenteric vessels
Greater omentum	Transverse colon	Apron anterior to small bowel	Epiploic vessels
Transverse mesocolon	Transverse colon	Pancreas	Middle colic vessels, gastrocolic trunk
Ascending mesocolon	Ascending colon	Mesenteric root	Marginal, ileocolic and right colic vessels
Descending mesocolon	Descending colon	Left superior duodenal fold	Marginal and left colic vessels, inferior mesenteric vein
Sigmoid mesocolon	Sigmoid colon	Root at origin of inferior mesenteric artery	Marginal and sigmoid vessels

### Anatomic continuity between the pararenal spaces

**Fig. 4:**
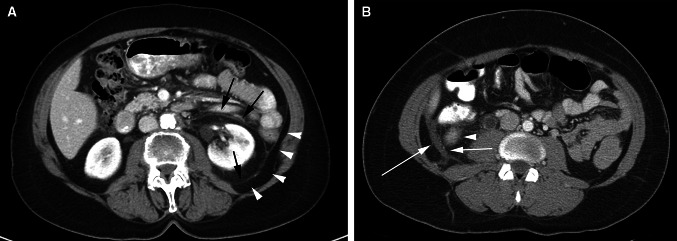
Renal fasciae. **A** Axial CT image shows the anterior and posterior renal fascia (*black arrows*). Arrowheads show the posterior pararenal space extending laterally as the properitoneal fat. **B** Axial CT image shows fluid (*arrows*) in between the 2 layers of the posterior renal fascia. Fluid from the anterior pararenal space can extend into this potential space between the 2 layers. *Arrowhead* points to the lower pole of the kidney.

The posterior portion of the extraperitoneal space, the retroperitoneum, in the abdomen is divided by the renal fascia into the anterior pararenal, perinephric, and posterior pararenal spaces (Fig. 4). The anterior pararenal space is between the parietal peritoneum and the anterior renal fascia; the perinephric space is between the anterior and posterior renal fascia; the posterior pararenal space is between the posterior renal fascia and the transversalis fascia. The posterior renal fascia has 2 layers. An anterior layer that is continuous with the anterior renal fascia and a posterior layer that forms the lateroconal fascia which in turn goes anterolaterally to blend with the peritoneum.

Laterally at the level of the kidney, the lateroconal fascia separates the anterior pararenal space from the posterior pararenal space. Below the level of the kidney and the iliac crest, the anterior and posterior renal fasciae tend to fuse resulting in anatomic continuity between the anterior and posterior pararenal spaces continuing inferiorly as the infrarenal space.

The anterior pararenal space contains the pancreas, duodenum, and ascending and descending colon. The perinephric space contains the kidney and adrenal gland. The posterior pararenal space has no organs and is continuous laterally with the extraperitoneal fat of the properitoneal flank stripe.

### Compartmentalization of the pelvic subperitoneal tissues

**Fig. 5:**
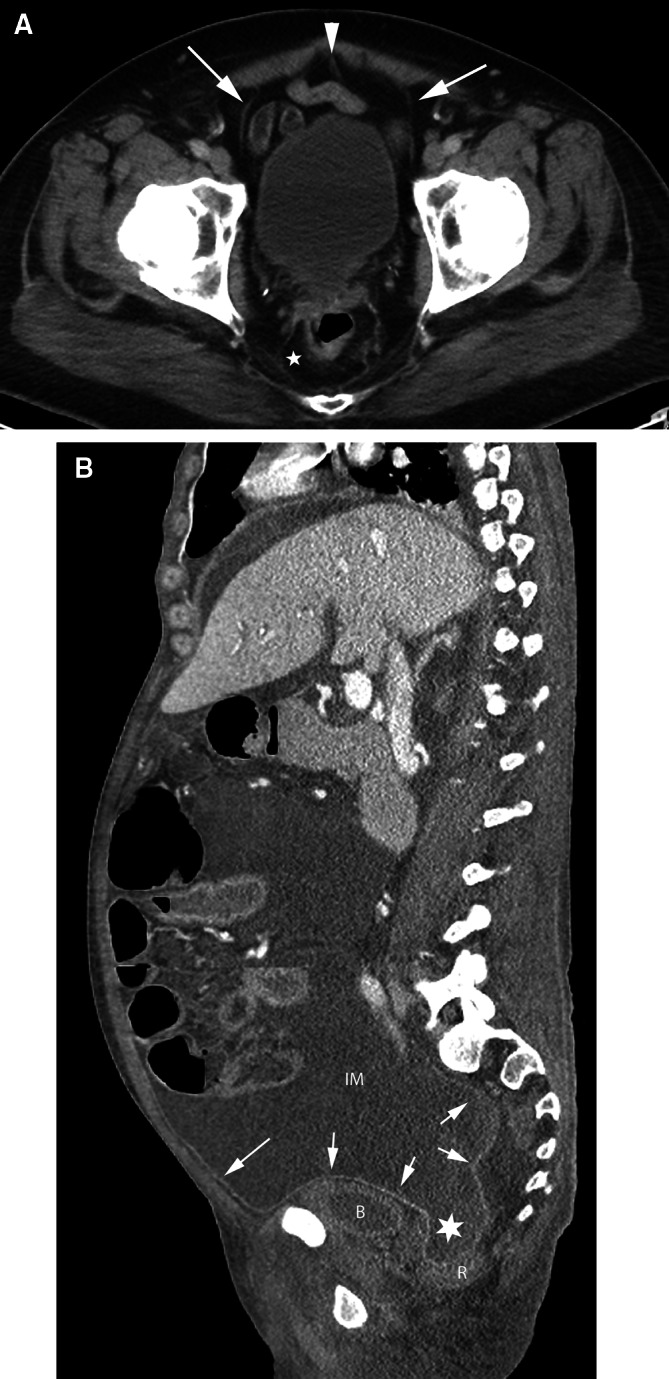
Pelvic spaces. **A** axial CT image of the pelvis. The urachus (*arrowhead*) and obliterated umbilical arteries (*arrows*) form the median umbilical ligament and the medial umbilical folds, respectively, and are encased within the umbilicovesical fascia forming the perivesical space. The prevesical space is anterior and lateral to the bladder. The perirectal space (*asterisk*) surrounds the rectum. **B** Sagittal CT image shows the peritoneum (*arrows*) along the anterior abdominal wall and reflecting over the bladder and rectum. Peritoneal fluid in the inframesocolic compartment (IM) communicates with the rectovesical recess (*asterisk*). The bladder is inferiorly displaced by the peritoneal fluid. *B* bladder, *R* rectum.

Anteriorly, the umbilicovesical fascia encloses the bladder and urachus in the perivesical space (Fig. 5). The umbilicovesical fascia also defines the prevesical space anteriorly and laterally. Posteriorly, the rectum is in the perirectal space which is defined laterally by the perirectal fascia, posteriorly by the posterior pelvic fascia, and anteriorly by the rectovesical fascia in the male and the rectovaginal fascia in the female. The aggregate of these fasciae is also referred to as the mesorectal fascia. Superiorly, the perirectal space communicates with the sigmoid mesocolon. The presacral space is posterior to the perirectal space.

The peritoneum reflects over the dome of the bladder, the uterine body, and the rectouterine recess (Fig. 5). Laterally, in the female, the peritoneum forms the folds of the broad ligament. In the female, the broad ligament suspends the female pelvic organs and is in continuity with the extraperitoneal space. Regions within the broad ligament form the mesometrium, mesosalpinx, and the mesovarium. These mesenteries are all in continuity within the broad ligament. The serosa of the fallopian tube is continuous as part of the broad ligament and its fimbriated end is open to the peritoneal cavity. The ovary lies within the broad ligament immediately beneath the peritoneum. The arteries, veins, lymphatics, and nerves to the female pelvic organs all course within the broad ligament and connect with the extraperitoneal space. This forms the pathways for the subperitoneal spread of disease.

## Overview of disease spread

### The pathways of disease spread in the abdominal cavity

**Fig. 6:**
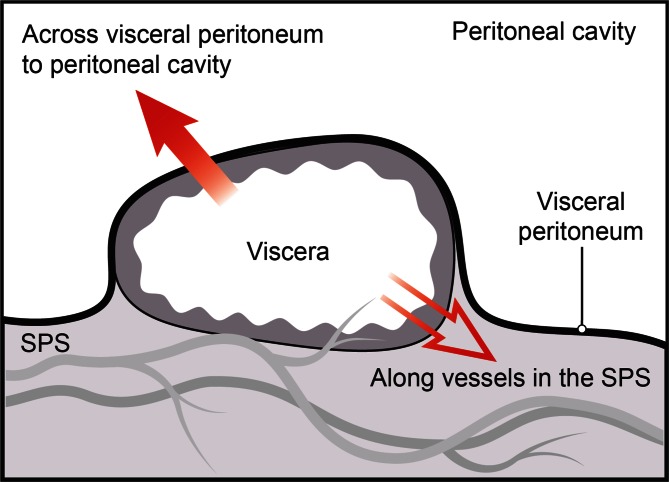
Diagram shows the 2 routes of disease spread for viscera which are covered by peritoneum–transperitoneal and subperitoneal. Disease can cross the visceral peritoneum (*solid arrow*) to enter and subsequently spread in the peritoneal cavity. Alternatively, disease can follow the vessels and lymphatics (*open arrow*) of the viscera to spread in the subperitoneal space (SPS).

Since all organs are subperitoneal, the subperitoneal space is a natural pathway for disease spread (Fig. 6). Potential routes are along the mesenteries and ligaments, via visceral lymphatics to nodes, and by periarterial, perineural, or transvenous routes as well as along ducts. Continuity of the subperitoneal space explains the spread of disease from one organ to another. Disease can spread bidirectionally within the subperitoneal space using the pathways created by normal structures. If a tumor is present, tracing the organ’s blood supply helps identify nodes that are potential pathways for spread. If an abnormal node is discovered, knowing its location helps in the search for the primary site.

For organs which are covered by peritoneum, there is an additional pathway for spread (Fig. 6). Transperitoneal spread occurs when disease traverses the visceral peritoneum along the surface of the organ. This route is possible because the peritoneum consists of a single layer of mesothelial cells on a bed of loose connective tissue [2]. Neoplastic and inflammatory cells, gas, and hemorrhage in the subperitoneal space can therefore cross the peritoneum to enter the peritoneal cavity. Subsequently, peritoneal spread occurs via the circulating peritoneal fluid to the peritoneal recesses in the abdomen and pelvis.

In summary, the pathways of disease spread are subperitoneal and peritoneal. In addition, transperitoneal spread occurs when subperitoneal disease crosses the peritoneal lining and spreads within the peritoneal cavity.

### Omental tumor: subperitoneal disease or peritoneal carcinomatosis

The surface of the greater omentum is lined by visceral peritoneum and the internal composition is of subperitoneal fat, lymphatics, and vessels. Communicating channels allow fluid in the peritoneal cavity to be absorbed by the omental lymphatics. Thus, tumor cells that are in the peritoneal cavity being disseminated by the peritoneal route can traverse the visceral peritoneum to grow in the richly vascular subperitoneal tissues of the omentum. In addition to the omentum, common sites of peritoneal tumor implants are the diaphragm and the dependent peritoneal recesses.

### Peritoneal fluid flow in the abdomen and pelvis

**Fig. 7:**
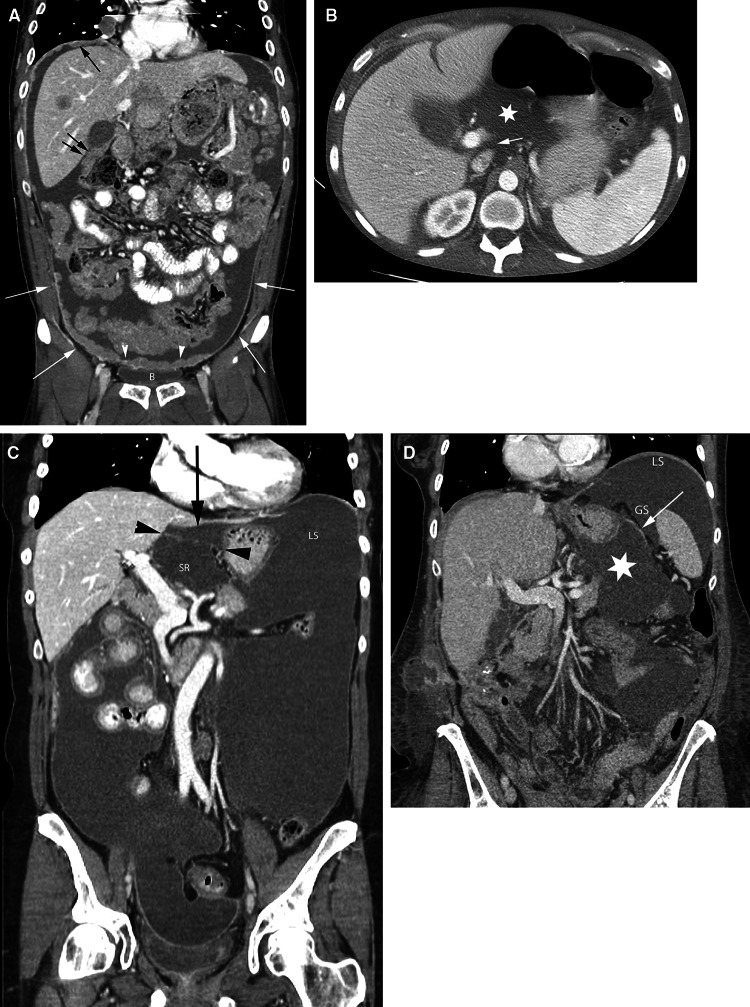
Peritoneal cavity fluid flow and recesses. **A** coronal CT image shows peritoneal carcinomatosis demonstrating the peritoneal recesses. Tumor in the right subdiaphragmatic recess (*black arrow*) and Morison’s pouch (*double black arrows*). Tumor (*arrowheads*) is also seen along the peritoneal reflection over the bladder (B). White arrows show continuity between the paravesical and paracolic recesses. **B** Axial CT image in a different patient shows the epiploic foramen (*arrow*) between the portal vein and inferior vena cava and fluid in the lesser sac (*asterisk*). **C** coronal CT image in a different patient shows fluid in the superior recess of the lesser sac (SR) and in the gastrohepatic recess (*black arrow*) separated by the gastrohepatic ligament (*black arrowheads*). Fluid in the gastrohepatic recess communicates with fluid in the left subphrenic space (LS). **D** coronal CT image in a different patient shows fluid in the lesser sac (*asterisk*) and in the gastrosplenic recess (GS) separated by the gastrosplenic ligament (*arrow*). There is continuity between the gastrosplenic and left subphrenic (LS) recesses.

The transverse mesocolon divides the peritoneal cavity into the supramesocolic and inframesocolic compartments. The latter is divided into right and left infracolic recesses by the root of the small bowel mesentery. Peritoneal fluid is drawn into the upper abdomen by low subdiaphragmatic pressures and is pulled into the pelvis under the influence of gravity. Fluid travels from the pelvis to the abdomen via the paracolic gutters and travels from the abdominal infracolic compartment to the pelvis (Fig. 7). It pools in dependent recesses, the most prominent of which are the pouch of Douglas in the female and the rectovesical recess in the male, along the superior portion of the sigmoid mesocolon, ileocolic region, right paracolic gutter, and Morison’s pouch.

All the peritoneal recesses communicate, however, peritoneal fluid preferentially flows in certain directions and is anatomically limited in some locations. Peritoneal fluid from the pelvis primarily goes up the right paracolic gutter (recess) forming continuity of the inframesocolic and supramesocolic recesses on the right. On the left, the phrenicocolic ligament limits the left paracolic gutter (recess) to the inframesocolic recess. From the right paracolic gutter, fluid enters the right subhepatic space (Morison’s pouch) and may subsequently enter the lesser sac via the epiploic foramen (of Winslow) between the main portal vein and the inferior vena cava (Fig. 7). Fluid also goes superiorly into the right subphrenic space but the falciform ligament limits flow from the right to the left subphrenic space. Abscesses secondary to intraperitoneal infections are therefore common in the pouch of Douglas, right paracolic gutter, right subhepatic space, and right subphrenic space. Fluid flow patterns are mostly bidirectional.

Although the falciform and phrenicocolic ligaments typically limit fluid flow across them, large volumes of fluid can overflow under the free edge of the falciform ligament and over the phrenicocolic ligament. Left subphrenic fluid is more commonly seen due to gastric, splenic, or splenic flexure colonic pathology. There is continuity between the left subphrenic space, the gastrohepatic space, and the perisplenic spaces such as the gastrosplenic recess and splenorenal recess. These are separated from the lesser sac by the gastrohepatic, gastrosplenic, and splenorenal ligaments, respectively.

### Distinguishing intraperitoneal and extraperitoneal fluid in the pelvis

**Fig. 8:**
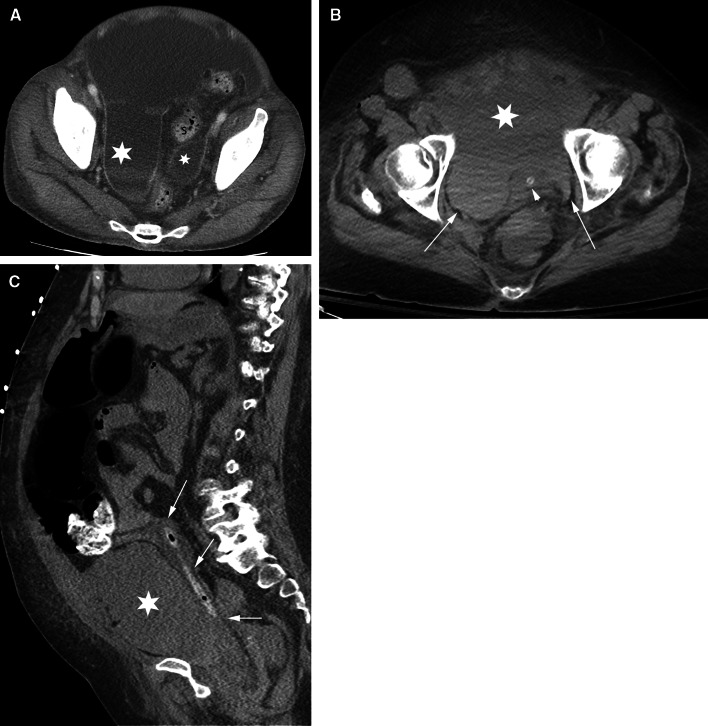
Pelvic intraperitoneal fluid vs extraperitoneal fluid. Pelvic fluid is shown in 2 different patients. The patient in **A** has intraperitoneal fluid. The patient in **B** and **C** has extraperitoneal fluid. **A** axial CT image shows intraperitoneal fluid in the right and left paravesical recesses (*asterisks*) lateral to the sigmoid colon (S) and superior to the bladder (not shown). **B** Axial CT image of a different patient shows extraperitoneal fluid in the prevesical space (*asterisk*) anterior to the decompressed bladder (*arrowhead*) containing a catheter. The bladder is displaced posteriorly. Fluid extends within the prevesical space (*arrows*) lateral to the bladder giving a “molar tooth” appearance. **C** sagittal CT image in the same patient as in B shows extraperitoneal fluid in the prevesical space (*asterisk*) anterior to the posteriorly displaced bladder (*arrows*) containing a catheter.

Intraperitoneal pelvic fluid (ascites) occurs in the pouch of Douglas and in the lateral recesses which lie on either side of the sigmoid colon and are referred to as paravesical recesses of the peritoneal cavity (Fig. 8). Extraperitoneal pelvic fluid in the subperitoneal space occurs in the prevesical space which is anterior and lateral to the bladder. Since the bladder is positioned more inferiorly in the pelvis than the sigmoid colon, ascites is seen superior to the bladder. Ascites displaces the bladder inferiorly, while prevesical space fluid displaces the bladder posteromedial. Extraperitoneal fluid in the prevesical space extends bilaterally forming a symmetric or asymmetric “molar tooth” appearance of fluid both anterior and lateral to the bladder (Fig. 8).

Ascites preserves the properitoneal fat which is the lateral extension of the posterior pararenal space and outlines the medial umbilical folds anteromedial. Extraperitoneal fluid in the prevesical space can extend to the lower abdomen and obliterate the properitoneal fat. The anterior extension spares the midline triangle of the perivesical fat that encases the urachus (median umbilical ligament) and the obliterated umbilical arteries (medial umbilical ligament). If prevesical fluid penetrates the overlying transversalis fascia, it can involve the rectus muscle. Conversely, rectus hematomas can extend into the prevesical fat.

Ascites travels superiorly from the lateral paravesical peritoneal recesses to the paracolic gutters. Prevesical fluid travels superiorly into the infrarenal space and subsequently into the pararenal spaces. Posteriorly, extraperitoneal fluid extends into the presacral space. Ascites can go into the inguinal canal along a hernia, while prevesical fluid can go to the inguinal ring along the vas deferens. Unlike ascites, prevesical fluid abuts the lateral pelvic musculature and can extend along the external iliac vessels and femoral sheath.

### Distinguishing intraperitoneal and extraperitoneal free air in the abdomen

The shape of the subdiaphragmatic air and change with respiration and position help distinguish intraperitoneal and extraperitoneal free air. On an upright chest radiograph, free intraperitoneal air follows the contour of the dome of the hemidiaphragm, while extraperitoneal air is usually medial or lateral to the apex of the hemidiaphragm. The volume of free intraperitoneal air seen under the hemidiaphragm increases on inspiration due to decreased subdiaphragmatic pressure. The volume of free extraperitoneal air seen under the hemidiaphragm increases on expiration due to decreased compression by the diaphragm. Free intraperitoneal air fills the potential recesses of the peritoneal cavity and can outline ligaments such as the falciform ligament. Free extraperitoneal air can occupy extraperitoneal spaces and can outline the psoas muscle and follow the flank stripe if within the posterior pararenal space. Peritoneal air shifts readily with position change, while extraperitoneal air does not.

### Potential routes of spread of extraperitoneal free air

Extraperitoneal free air originating anywhere in the abdomen can spread throughout the abdomen and pelvis via the interconnecting subperitoneal space.

Air from a duodenal perforation can go from the anterior pararenal space to the infrarenal space and then to the posterior pararenal space or to the prevesical space of the pelvis.

**Fig. 9:**
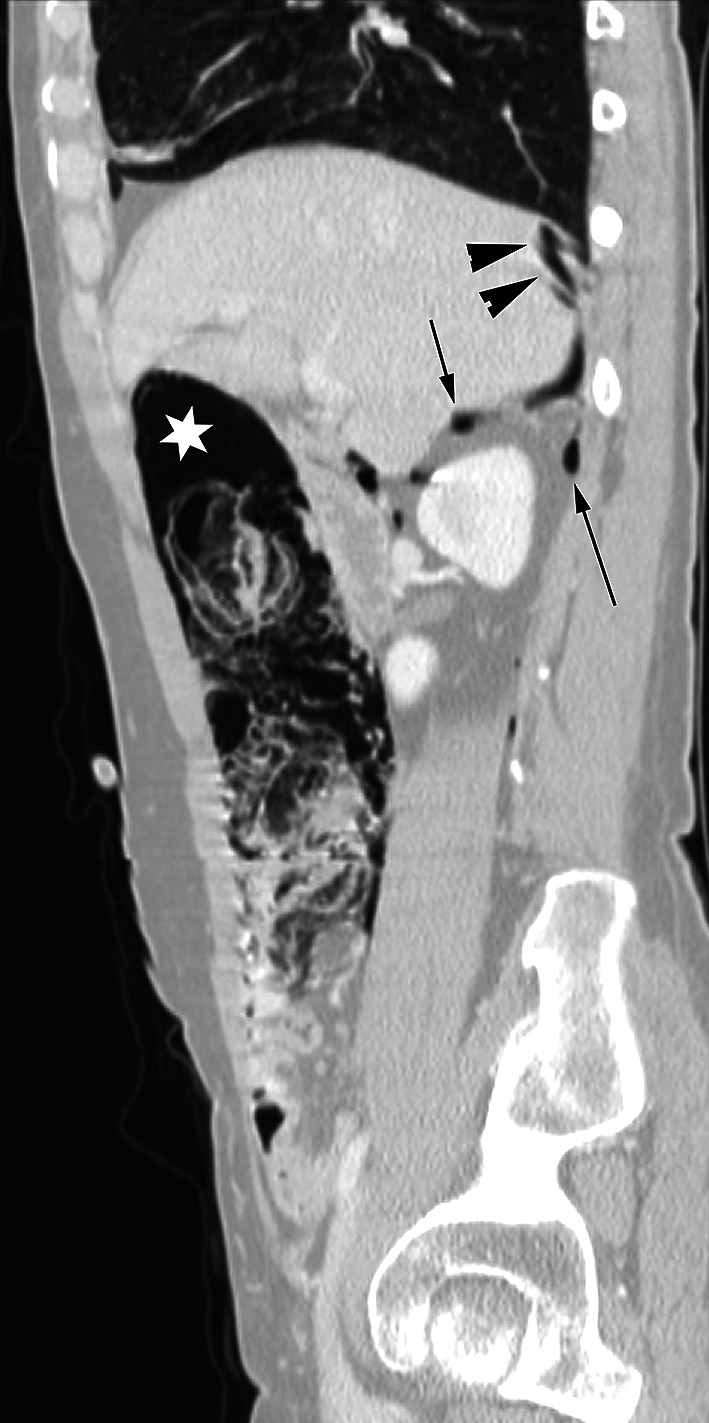
Extraperitoneal free air. Sagittal CT image shows pneumatosis of the ascending colon and extraluminal air in the adjacent mesentery (*asterisk*). This extraperitoneal free air tracks in the anterior and posterior pararenal spaces (*arrows*) to the bare area of the liver and diaphragm (*arrowheads*).

Extraperitoneal pelvic free air from a rectal perforation can go from the mesorectum to the sigmoid mesocolon to the prevesical space. From the prevesical space air can go inferiorly along the vas deferens to the scrotum, laterally outside the pelvic cavity via the sciatic foramen and into the thighs along the scaffold of the femoral sheath. From the prevesical space air can also go superiorly into the infrarenal space and then to the posterior pararenal space or to the anterior pararenal space. Extraperitoneal air in the posterior pararenal space can extend laterally within the properitoneal fat and superiorly to the level of the respiratory diaphragm (Fig. 9). In the anterior pararenal space air can surround the pancreas and extend to the porta hepatis, to the bare areas of the liver and spleen, and to the root of the mesenteries of the small bowel and transverse colon. Continuity within the anterior pararenal space allows for bidirectional spread between the organs of the extraperitoneum and suspended organs via their mesenteries all within the subperitoneal space.

Extraperitoneal air can also track along the aorta and into the chest through the aortic hiatus. Extraperitoneal air in the gastrohepatic ligament can enter the chest through the esophageal hiatus. This spread is bidirectional and air from the mediastinum can spread throughout the abdomen within the subperitoneal space.

## Disease spread for selected organs

### Potential routes of spread of pancreatic disease

**Fig. 10:**
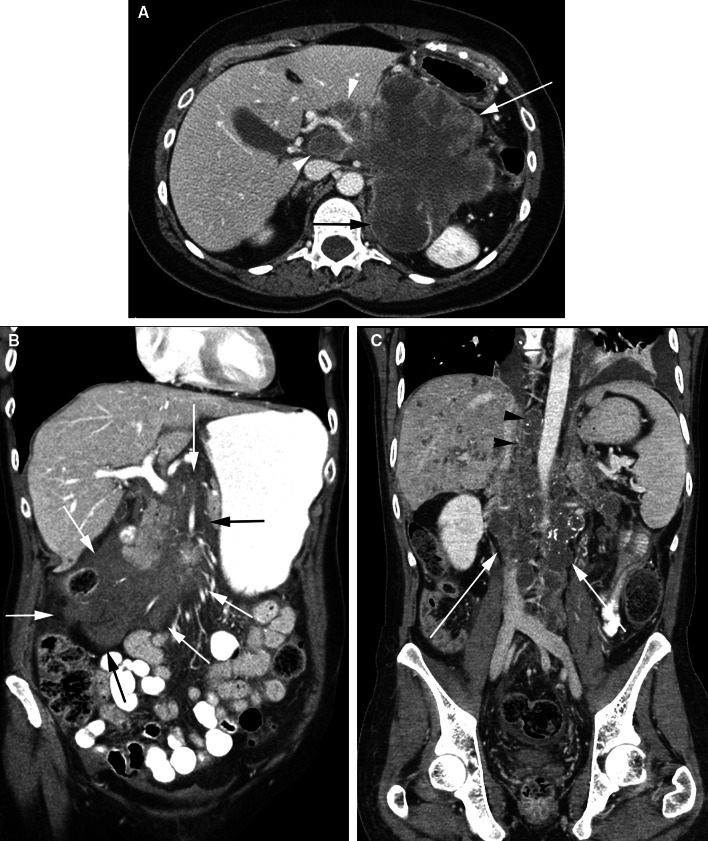
Subperitoneal spread of disease. **A** Axial CT image shows a large pancreatic mass (*white arrow*) extending along the hepatoduodenal ligament to the porta hepatis (*arrowheads*). Mass also invades the left perinephric space and engulfs the adrenal gland (*black arrow*). Superiorly, the mass extended into the gastrohepatic ligament with invasion of the left lobe of the liver (not shown). **B** Coronal CT image shows a hematoma in the root of the small bowel mesentery extending toward the ileocecal junction within the small intestine mesentery (*arrows*). **C** Coronal CT image shows a heterogeneous mass (*white arrows*) around the inferior vena cava and aorta. Mass extends superiorly, through the aortic hiatus, to the posterior mediastinum (*black arrowheads*). The mass also extended into the root of the small bowel mesentery (not shown) along the superior mesenteric artery.

The spread of pancreatic disease highlights the concept of the subperitoneal space as a single space (Fig. 10). The pancreas lies in the anterior pararenal space. This space is a natural pathway as it connects with the base of several mesenteries. The tail of the pancreas lies in the splenic hilum so disease can spread along the gastrosplenic ligament to the gastrocolic ligament along the greater curvature of the stomach. The head of the pancreas is connected to the hepatic hilum by the hepatoduodenal ligament so disease can spread along the gastrohepatic ligament to the lesser curvature of the stomach. The root of the transverse mesocolon lies along the pancreas so disease can spread to the transverse colon. Similarly, the root of the small bowel mesentery starts adjacent to the anterior inferior aspect of the pancreatic body so disease can spread to the ileocolic region.

The peritoneum forming the posterior margin of the lesser sac is anterior to  the pancreas. Disease can cross the peritoneum (transperitoneal spread) to enter the lesser sac for subsequent peritoneal spread.

### Anatomic basis for a hepatic laceration resulting in a retroperitoneal hematoma

**Fig. 11:**
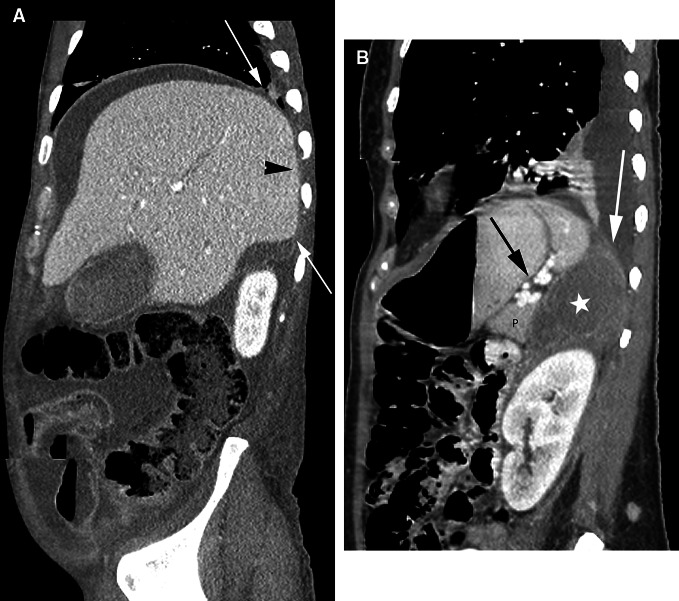
Extraperitoneal spaces in the upper abdomen. **A** Sagittal CT image shows the peritoneal reflections over the liver (*arrows*). The liver posterior and medial to these reflections is left bare of peritoneum and abuts the diaphragm (*arrowhead*). Note that peritoneal fluid surrounds the liver except for the bare area. **B** Sagittal CT image shows fluid in the left perinephric space (*asterisk*) extending superiorly to abut the diaphragm (*white arrow*). The fluid is posterior to the pancreas (P) and the splenorenal ligament (*black arrow*).

The bare area of the liver is that part of the posterior right lobe of the liver that is not covered by peritoneum (Fig. 11). The peritoneum covering the liver reflects on itself to form the right coronary ligament and attach the right lobe to the right hemidiaphragm. The liver posterior and medial to this reflection is left bare of peritoneum and abuts the diaphragm. In addition, the parietal peritoneum of the inferior right coronary ligament also fuses with the anterior renal fascia. Posteriorly, the diaphragmatic fascia fuses with the right posterior renal fascia. As a result, the space between the anterior and posterior renal fascia, or the right perinephric space, communicates with the bare area of the liver. Hemorrhage from the hepatic bare area can therefore present as a retroperitoneal bleed in the right perinephric space. Similarly, a right perinephric abscess can directly spread to the liver and diaphragm.

On the left, the perinephric space communicates with the left subphrenic extraperitoneal space (Fig. 11) [2]. This is the result of the left anterior renal fascia fusing with the gastrosplenic ligament and the posterior renal fascia fusing with the diaphragm [2]. In addition, the peritoneum covers most of the spleen except for the splenic hilum. The splenorenal ligament connects the bare area of the spleen at the hilum with the left anterior pararenal space. Bleeding from a ruptured splenic artery aneurysm can therefore result in a retroperitoneal bleed in the anterior pararenal space.

### Anatomic basis for a hepatobiliary tumor mimicking a pancreatic mass

Tumor from the liver and gallbladder can spread to the nodes within the hepatoduodenal ligament. The anterior periportal nodes follow the lymphatics along the hepatic artery to the celiac artery and then to the cisterna chyli. The posterior periportal nodes follow the lymphatics to the retropancreatic nodes, aortocaval node, and then to the cisterna chyli. Therefore, an enlarged retropancreatic node from a metastatic hepatobiliary tumor can mimic a pancreatic mass. Other routes of nodal spread are the gastrohepatic ligament or adjacent to the suprahepatic inferior vena cava to the juxtaphrenic and paraesophageal nodes.

### Diaphragmatic adenopathy secondary to peritoneal carcinomatosis

Similar to the omentum, the diaphragm has abundant lymphatics for the absorption of peritoneal fluid. This process can result in tumor cells being transported from the peritoneal cavity to the diaphragmatic nodes. Metastatic diaphragmatic adenopathy can therefore occur secondary to peritoneal carcinomatosis from abdominal and pelvic malignancies.

### Potential routes of peritoneal spread of blood following trauma

Traumatic injuries of viscera can disrupt the capsule of an organ. The visceral peritoneum forms the capsule of the liver and spleen, except for the bare areas described earlier. Therefore, a laceration allows for transperitoneal spread of blood from these organs into the peritoneal cavity. Following a laceration of the peritonealized surface of the liver, blood can spread into the right subphrenic space and right subhepatic space and can subsequently enter the lesser sac and right paracolic gutter. Following a laceration of the peritonealized surface of the spleen, blood can enter the perisplenic space, left subphrenic space, and gastrohepatic space. Larger volumes can also spread to the left paracolic gutter. From the paracolic gutters, blood can accumulate in the dependent rectovesical recess of the pelvis.

Since the peritoneum forms the serosa of bowel, bowel perforations can result in blood, air and bowel contents such as endoluminal contrast spilling into the peritoneal cavity. The mesenteric vessels lie in subperitoneal fat between 2 layers of visceral peritoneum. Blood from a mesenteric hematoma can therefore traverse the visceral peritoneum to enter the peritoneal cavity and appear as intraperitoneal hemorrhage. Similarly, hemorrhage in tissues around extraperitoneal organs such as the pancreas and kidney can cross the posterior peritoneum to enter the peritoneal cavity.

In the pelvis, the peritoneum reflects over the bladder dome. A rupture of the bladder dome results in intraperitoneal spill of urine and administered bladder contrast into the paravesical, paracolic, and inframesocolic recesses. Intraperitoneal bladder rupture is less common than extraperitoneal bladder rupture.

At any site in the abdomen and pelvis, once blood, air, or contrast has entered the peritoneal cavity, it can travel to any of the peritoneal recesses.

## Summary

The two spaces of the abdomen are separated by the peritoneum (Table 1).

The peritoneal cavity is a potential space between the visceral and parietal layers of the peritoneum. This potential space normally contains only a small amount of peritoneal fluid and is not seen on transaxial imaging of normal patients. The peritoneal cavity is distinct from the subperitoneal space and contains no organs or structures. The spread pattern in the peritoneal cavity follows the flow of peritoneal fluid.

The subperitoneal space is a continuous interconnecting space beneath the peritoneum containing the extraperitoneal space, the ligaments and mesenteries, and their suspended organs. Individual mesenteries and ligaments are identified by the vessels that course through them. The subperitoneal space provides the avenues for bidirectional spread of disease.

Transperitoneal spread occurs when disease spreads from the subperitoneal space to the peritoneal cavity by crossing the peritoneal lining.
